# CT and MRI findings of cystic renal cell carcinoma: comparison with cystic collecting duct carcinoma

**DOI:** 10.1186/s40644-021-00419-1

**Published:** 2021-09-07

**Authors:** Qingqiang Zhu, Jun Ling, Jing Ye, Wenrong Zhu, Jingtao Wu, Wenxin Chen

**Affiliations:** Department of Medical Imaging, Clinical Medical College, Yangzhou University, No 98 West Nantong Road, 225001 Yangzhou, China

**Keywords:** Cystic renal cell carcinoma, Cystic collecting duct carcinoma, Differential diagnosis, Computerized tomography

## Abstract

**Background:**

Cystic renal cell carcinoma (CRCC) and cystic collecting duct carcinoma (CCDC) share similar oncogeni and some imaging findings. The aim of this study was to characterize the clinical and CT imagings features of CRCC and CCDC.

**Methods:**

Thirty-three patients with CRCC and thirteen patients with CCDC with pathologically proven were retrospectively studied. Tumor characteristics were assessed.

**Results:**

On CT imaging, 33 patients(100 %) with CRCC and 13 patients(100 %) with CCDC, tumors calcifications (8 vs. 9, *P* < 0.0001), had a clear boundary (capsule sign, 30 vs. 2, *P* < 0.0001), infiltrative appearance (1 vs. 13, *P* < 0.0001), exogenous appearance (29 vs. 3, *P* < 0.0001), invaded the renal pelvis or ureter (1 vs. 10, *P* < 0.0001), hemorrhage (1 vs. 10, *P* < 0.0001), had retroperitoneal lymph node or distant metastasis (2 vs. 10, *P* < 0.0001), thickened enhancing internal septations (31 vs. 2, *P* < 0.0001), and mural soft-tissue nodules (21 vs. 1, *P* < 0.0001). On MR imaging,13 patients(39 %) with CRCC and 4 patients(31 %) with CCDC, all CRCCs appeared hypointense on T1-weighted images and hyperintense on T2-weighted images, however, all CCDCs appeared hypointense on T1-weighted images and hypointense on T2-weighted images(*P* < 0.0001). 33 patients with CRCC, they were all alive from3 years to 10 years follow-up, however, 13 patients with CCDC, of which 11 patients were able to be followed up, and 9 patients expired within 5 years of the initial diagnosis and the others are currently still alive.

**Conclusions:**

Distinguishing features of CRCC and CCDC included calcifications, capsule signs, infiltrative appearance, metastasis, internal septations, mural nodules and signal on CT or MR images. These imaging features may help in differentiating the two renal tumor types.

## Background

Cystic renal cell carcinoma (CRCC) composed entirely of numerous cysts, the septa of which contain small groups of clear cells indistinguishable from grade Ι clear cell carcinoma [1]. Collecting duct carcinoma is rare, accounting for < 1 % of renal malignancies, especially for cystic tumors [2]. It is interesting that CRCC and cystic collecting duct carcinoma (CCDC) share similar oncogenic, histologic features and some imaging findings [3]. No instance of progression of CRCC is known and no tumour with these features has ever recurred or metastasized. However, the CCDCs have a poor prognosis with many being metastatic at presentation. About two-thirds of CCDC patients die of their disease within two years of diagnosis [3]. A radical operation might be regarded as an over-treatment for CRCC, with nephron-sparing surgery (partial nephrectomy) being a more appropriate alternative. Nephron-sparing surgery has been recommended for CRCC by many authors, with increasingly many studies confirming good long-term results and excellent patient survival [4]. However, a radical operation is advocated by many surgeons for CCDC. So, an accurate diagnosis is important for guiding clinical treatment. Therefore, the aim of this study was to characterize the clinical and CT imagings features in 33 cases of CRCC and 13 cases of CCDC.

## Methods

### Dataset

This study was approved by our institutional review board with a waiver of the requirement for written informed consent. Exclusive criteria included: (1) patients who received previous treatment or experienced postoperative recurrence of CD C; (2) the diameter of the mass in an axial plane less than 1 cm; (3) the mass was solid. A search of pathology records and PACS system identified 33 patients with CRCC and 13 cases of CCDC retrospectively who were hospitalized at our hospital between 2010 and 2020. Details of the patients age, gender, surgery or biopsy confirmation, metastasis, and clinical symptoms were recorded.

### CT imaging technique

CT was performed with one of two clinical scanners: a 16-MDCT scanner, and a 64-MDCT scanner (Lightspeed Ultra; GE Healthcare). Scanning parameters were 120kVp; 200–300mAs; section thickness, 3 mm; collimation, 0.6–2.5 mm; pitch, 1.0–1.4; reconstruction interval, 50 %; FOV, 33 cm; rotation time, 0.5 s; reconstruction kernel, B40. Images were obtained with a renal mass protocol that included unenhanced images followed by nephrographic phase images 120 s after IV administration of nonionic contrast medium (iohexol 350 mgI/mL, Omnipaque, GE Healthcare) at 3–4 mL/s and volume of 1.5 mL/kg followed by a 20-mL saline flush.

### Magnetic Resonance Imaging (MRI)

Thirteen cases of CRCC and four cases of CCDC also underwent unenhancement MRI. MRI examinations were performed with a 3.0-T MR scanner (GE 750, Milwaukee, WI, USA) using a 6 channel array body coil and a 24 channel phased array spine coil integrated into the scanner table. All sequences were acquired with anterior and posterior saturation bands. Axial T1-weighted (T1W) and T2-weighted (T2W) MR images were obtained during breath-hold.

### Pathologic examination

The histopathologic criteria included the macroscopic and microscopic aspects of the tumors. We examined microphotographs obtained with HE staining, in order to observe the cellular morphology and growth patterns of the tumors. Gross specimens were evaluated at the time of surgery for fibrous capsule formation, invasion into the renal calyx, pelvis or ureter, and invasion into the renal vein or inferior vena cava. Pathological specimens were observed by light microscopy and immunohistochemical analysis. All renal tumors were confirmed to be CRCC or CCDC.

### Imaging analysis and statistics

Two radiologists with more than 10 years’ experience each, blinded to the final diagnosis, reviewed the CT or MR images in consensus at a picture archiving and communication system workstation. Before image interpretation, the readers met and agreed on a scoring system to be used for cyst classification, and they designed a data collection sheet. Readers recorded the Bosniak cyst category in each case [5, 6]. After the independent interpretation sessions, a consensus reading was used for cases of disagreement as to cyst classification. The evaluated parameters included tumor calcifications, hemorrhage, signal, thickened enhancing internal septations and mural soft-tissue nodules. The presence of a capsule, retroperitoneal lymph nodes or other metastasis, perinephric stranding, hydronephrosis, and renal tissue invasion were also documented. Infiltrative growth on CT/MRI is characterized by poorly marginated borders between the tumor and normal renal parenchyma, whereas exogenous growth is characterized by well-defined bulging tumor margins that displace normal parenchyma. Internal septations: one or more complete or partial septations within the mass. Lymph node metastasis was indicated when a lymph node was enlarged more than 1.5 cm in diameter. Hemorrhage component was considered to be present if unenhanced attenuation > 45HU, non-enhancing was noted during CT enhancement.

Calcification component was considered to be present if unenhanced attenuation > 100HU.

### Statistical analysis

Statistical analyses were undertaken using SPSS 17.0 statistical software (SPSS Inc, Chicago, Illinois, USA). Numeric data were expressed as mean ± standard deviation and categorical data were expressed as percentages. Independent-samples t test was used to analyze normally distributed continuous data. Evaluated characteristics were compared using fisher’s exact test. Interobserver agreement of CT and MRI features was evaluated by using the intra class correlation coefficient (ICC) with 95 % confidence interval (CI) (≤ 0.20, slight; 0.21–0.40, fair; 0.41–0.60, moderate; 0.61–0.80, substantial; and 0.81–1.00, perfect). *P* < 0.05 was considered statistically significant.

## Results

### The clinical and CT or MR imaging features of CRCC and CCDC

The study included 33 patients (20 females and 13 males) with CRCC and 13 patients (7 males and 6 females) with CCDC. The mean age at diagnosis was slightly higher in patients with CRCC (52.1 years; range 43 to 68 years) than in those with CCDC (53.2 years; range 37 to 68 years, *P* > 0.05). Presenting symptoms of CRCC and CCDC included flank pain (26 vs. 12, *P* > 0.05), hematuria (5 vs. 11, *P* < 0.0001), palpable mass (9 vs. 3, *P* > 0.05) and fever (12 vs. 5, *P* > 0.05).

**Fig. 1:**
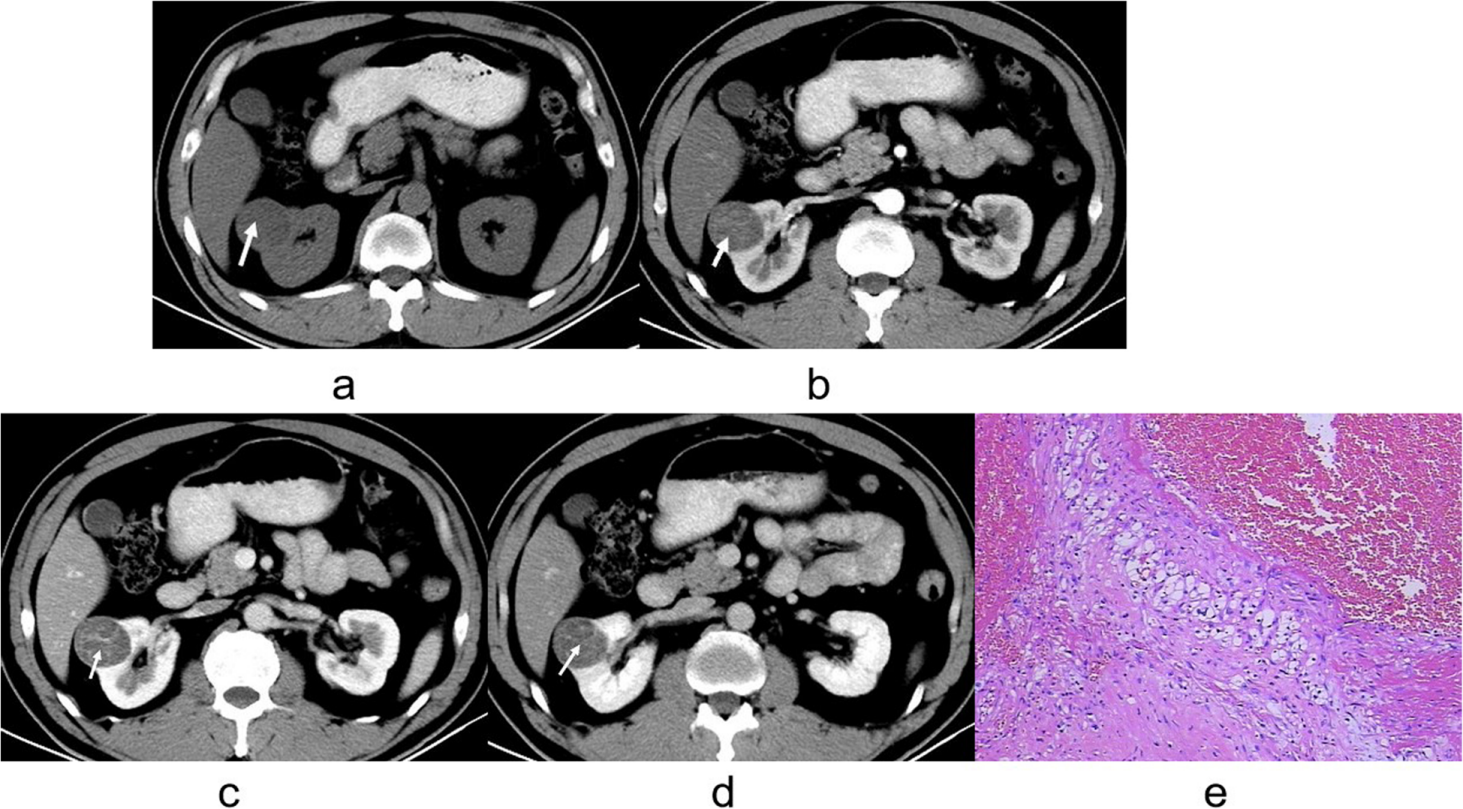
**a-e**. CRCC of right kidney. **a** Unenhanced CT scan showed a hypodense mass in the medulla, clear boundary, and showed an exogenous appearance (arrow). **b** Mild enhancement was noted on the cortex phase (arrow). **c, d** The tumors showed thickened enhancing internal septations on the medulla and delayed phases (arrow). **e** The tumor cells showed the lining cells ranges from clear to pale (H&E stain; original magnification, ×400)

**Fig. 2:**
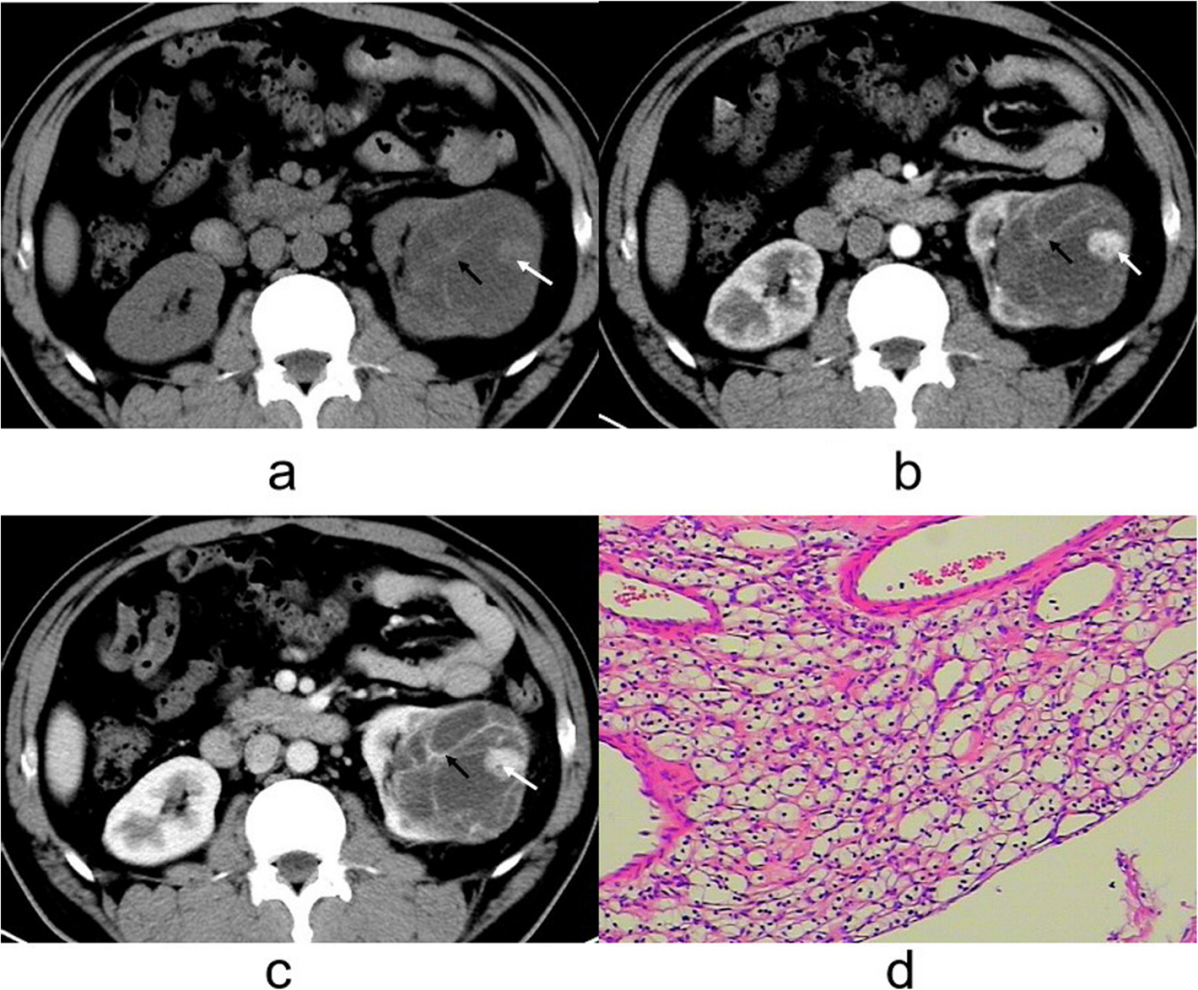
**a-d**. CRCC of left kidney. **a** Unenhanced CT scan showed a hypodense mass in the medulla, clear boundary, and showed an exogenous appearance (arrow). **b, c** Obvious enhancement was noted with mural soft-tissue nodules (white arrow) together with thickened enhancing internal septations (black arrow) on the cortex and medulla phases. **d** The tumor cells showed the cytoplasm ranges from clear to pale. The septa consist of fibrous tissue, densely collagenous(H&E stain; original magnification, ×400)

**Fig. 3:**
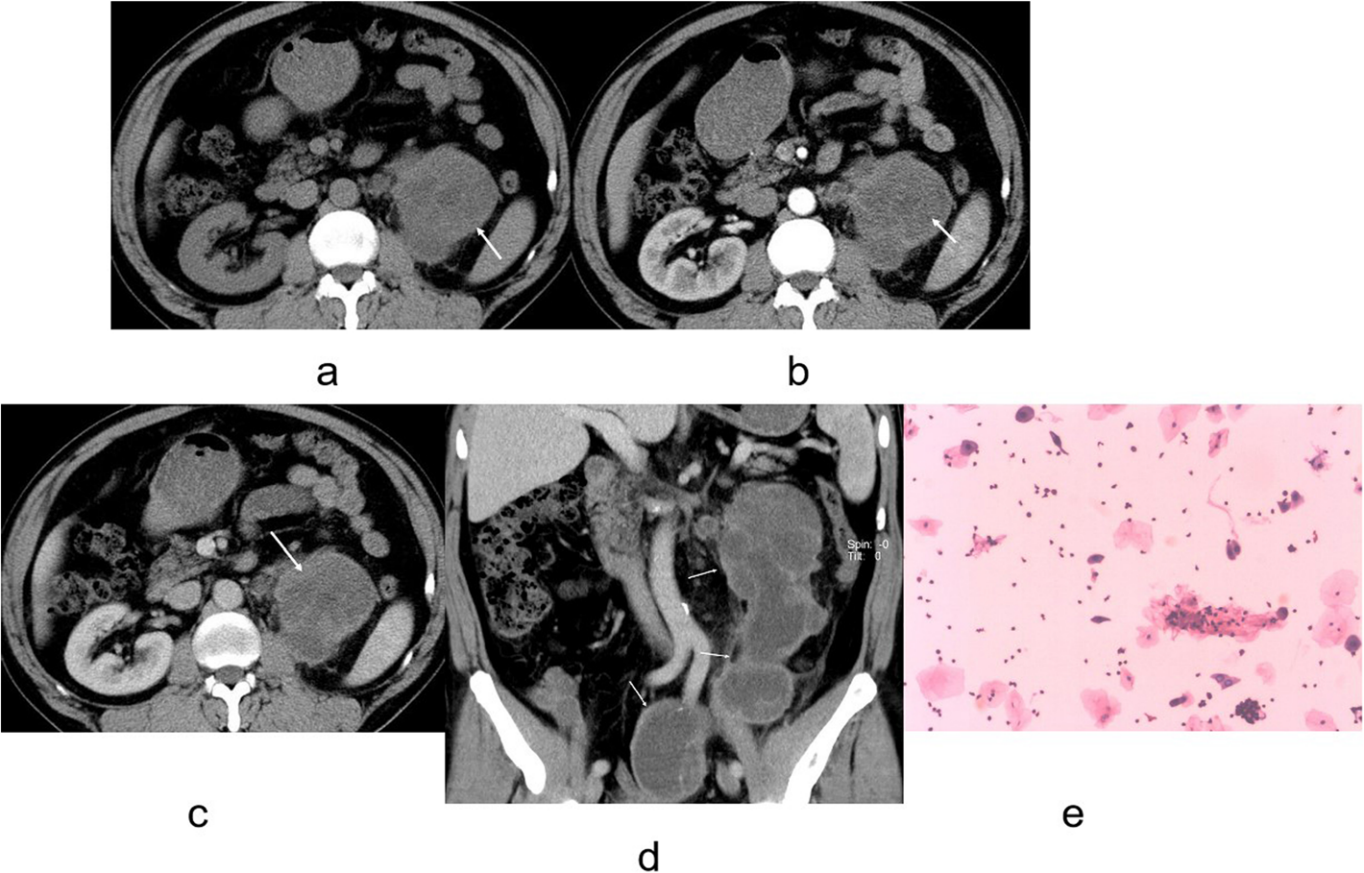
**a-e**. CCDC of left kidney. **a** Unenhanced CT scan showed a hypodense mass in the medulla, unclear boundary, and showed an infiltrative appearance (arrow). **b, c** Mild, heterogeneous enhancement was noted on the cortex and medulla phases (arrow). **d** The tumors invaded the renal pelvis ureter (arrows). **e** The tumors cells were tubular and papillary growth pattern (H&E stain; original magnification, ×400)

**Fig. 4:**
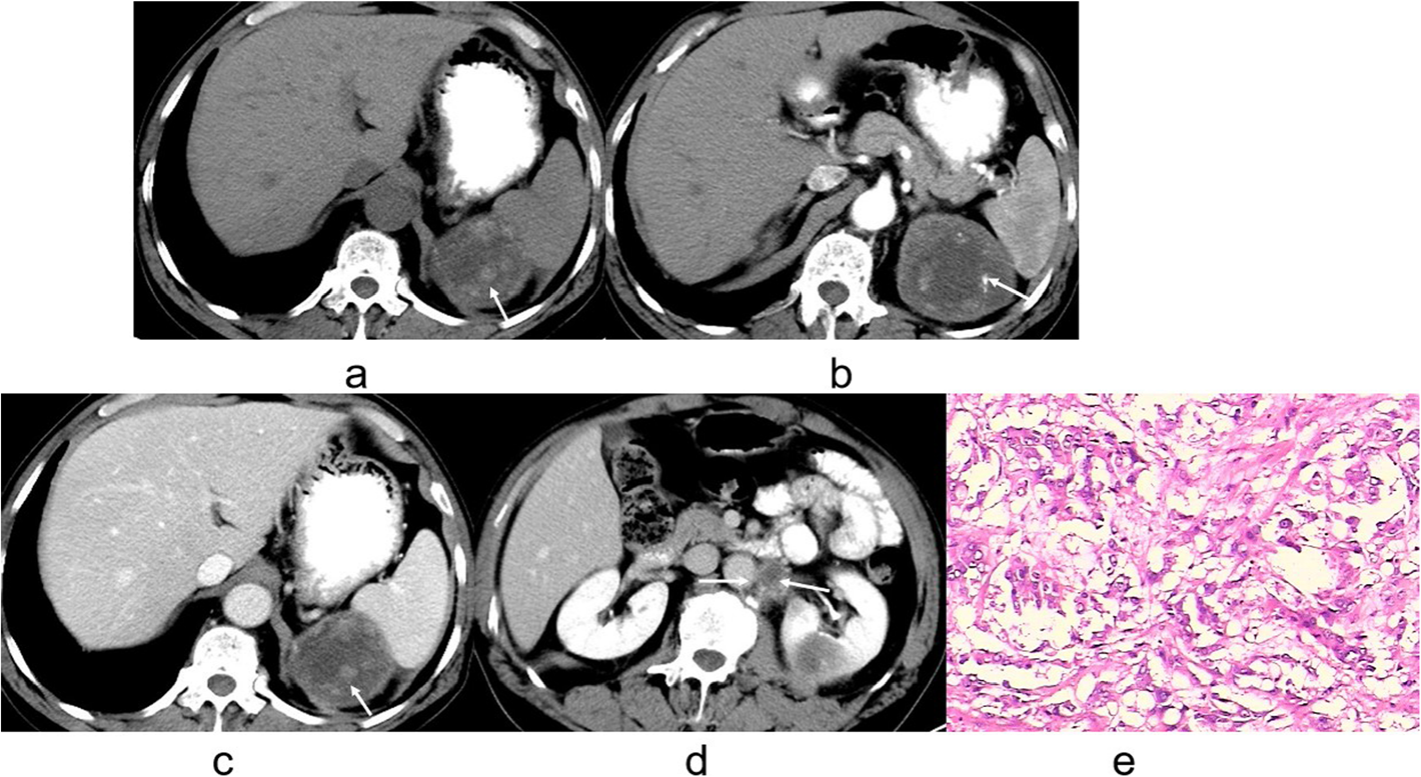
**a-e**. CCDC of left kidney. **a** Unenhanced CT scan showed a heterogeneous mass together with hemorrhage and calcification (arrow). **b, c** Mild, heterogeneous enhancement was noted on the cortex and medulla phases (arrow). Tumors had an unclear boundary and showed an infiltrative appearance (arrow). **d** Tumors had retroperitoneal lymph node metastasis (arrow). **e** The tumor cells showed tubulopapillary, pseudopapillary, and cribriform patterns (H&E stain; original magnification, ×400)

There was evidence of calcifications in 8 cases of CRCC whereas 9 patients with CCDC had evidence of calcifications (*P* < 0.0001, Table 1). In 30 patients with CRCC (Figs. 1 and 2) and in 2 with CCDC (Figs. 3 and 4) the tumors had a clear boundary (*P* < 0.0001, Table 1). Tumors showed an infiltrative appearance on CT in 13 cases of CCDC (Figs. 3 and 4), whereas only one case of CRCC showed an infiltrative appearance (*P* < 0.0001). An exogenous appearance was present in 29 of CRCC (Figs. 1 and 2), but an exogenous appearance was present in 3 of CCDC (*P* < 0.0001, Table 1). In one patient with CRCC and in 10 with CCDC (Fig. 3 ) the tumors invaded the renal pelvis or ureter (*P* < 0.0001). In 2 patients with CRCC and 10 patients with CCDC (Fig. 4d) had retroperitoneal lymph node or distant metastasis (*P* < 0.0001). Hemorrhage was found in 10 cases with CCDC whereas only one case was found in CRCC (*P* < 0.0001, Table 1). In 31 patients with CRCC (Figs. 1 and 2) and in 2 with CCDC the tumors had thickened enhancing internal septations (*P* < 0.0001). Mural soft-tissue nodules were found in 21 cases with CRCC (Fig. 2) whereas only one case was found in CCDC (*P* < 0.0001, Table 1).

**Table 1:** Comparative study of CT appearances in cystic renal cell carcinoma (*n* = 33) and cystic collecting duct carcinoma (*n* = 13)

Main CT findings	CRCC (*n* = 33)	CCDC (*n* = 13)	*P* value
calcification	8	9	0.004
capsule sign	30	2	<0.0001
infiltrative appearance	1	13	<0.0001
exogenous appearance	29	3	<0.0001
invaded renal pelvis/ ureter	1	10	<0.0001
metastasis	2	10	<0.0001
hemorrhage	1	10	<0.0001
internal septations	31	2	<0.0001
mural nodules	21	1	<0.0001

Note: *CRCC* cystic renal cell carcinoma, *CCDC* cystic collecting duct carcinoma

**Fig. 5:**
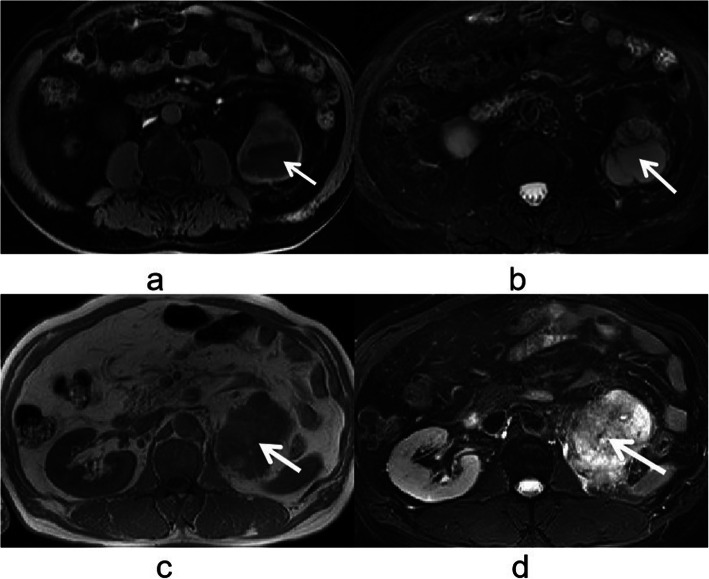
**a-d**. CRCC and CCDC of MR imaging. **a** CRCC appeared hypointense on T1-weighted images. **b** CRCC appeared hyperintense on T2-weighted images. **c** CCDC appeared hypointense on T1-weighted images. **d** CCDC appeared hypointense on T2-weighted images

On MR imaging, 13 patients with CRCC and 4 patients with CCDC, all CRCCs appeared hypointense on T1-weighted images (Fig. 5a) and hyperintense on T2-weighted images (Fig. 5b), however, all CCDCs appeared hypointense on T1-weighted images (Fig. 5c) and hypointense on T2-weighted images (Fig. 5d) (*P* < 0.0001).

### Interobserver agreement

The kappa value for the independent readings of the two radiologists was 0.926, perfect agreement.

### Follow-up

Thirty-three patients with CRCC underwent total nephrectomy(tumor grading, III/IV, *n* = 19) or local resectiony(tumor grading, I/II, *n* = 19) with a satisfactory outcome. No adjuvant therapy was given, and they were all alive without manifesting disease or any other signs or symptoms from3 years to 10 years follow-up.

In this study, all of the 13 patients with CCDC underwent radical nephrectomy, of which 11 patients were able to be followed up. 9 patients expired within 5 years of the initial diagnosis and the others are currently still alive.

### Pathologic findings

Microscopy sections of CRCC tumor from two different patients are shown in Figs. 1e and 2d. Microscopic analysis indicated that CRCC tumor cells showed the lining cells are flat or plump and their cytoplasm ranges from clear to pale. The septa consist of fibrous tissue, often densely collagenous.

Microscopy sections of CCDC tumor from two different patients are shown in Figs. 3e and 4e. Microscopic analysis indicated that CCDC tumor cells showed tubular, papillary, tubulopapillary, pseudopapillary, and cribriform patterns.

## Discussion

According to the 2004 World Health Organization classification of renal tumors, CRCC is a rare subtype of renal cell carcinoma. There is a male:female predominance of 3:1. All have been adults (age range 20–76 years, mean = 51). No instance of progression of CRCC is known [7].

Completely CCDCs are rare, accounting for < 1 % of renal malignancies. Over 100 cases have been described and there is a wide age range from 13 to 83 years (mean, about 55) with a male to female ratio of 2:1 [8]. It is interesting that CRCC and CCDC share similar oncogenic, histologic features and some imaging findings. Although CRCC and CCDC have been relatively well described in pathology studies, comparative study of CT appearances are scanty, particularly in combination with histopathologic examination. However, patients with CRCC have a better prognosis after undergoing total nephrectomy or local resection than that CCDC [9, 10]. So, an accurate diagnosis is important for guiding clinical treatment.

Histopathologycally, CCDC arise from the medulla as have been previously noted in the literatures, which distinguish them from the CRCCs that arise from the renal cortex [11]. Other tumors may also involve the renal medulla, e.g., transitional cell carcinoma [12], squamous cell carcinoma [13] and chromophobe renal cell carcinoma [14]. It is difficult to differentiate CCDC from other tumors if only relying on tumor position [15]. Other characteristics may be helpful, e.g., transitional cell carcinomas arise from the collecting system and may cause hydronephrosis [16]. Transitional cell carcinomas, also tend to involve the kidney by infiltration [17]. A centrally located CCDC with invasion into the renal pelvis may be indistinguishable from an invasive transitional cell carcinoma of the renal pelvis. This distinction, however, has important implications for treatment because nephroureterectomy is indicated for urothelial carcinomas, whereas nephrectomy is performed for renal parenchymal malignancies [18]. Chromophobe renal cell carcinomas may have a spoke-like pattern in some cases.

Our results show that 10 cases of CCDCs appear as heterogeneous hyper-attenuating tumors(> 45HU), while all cases of CRCCs appear as hypodense masses. Other authors reported that the pathological basis for hyperdense appearance of a tumor on unenhanced CT was mainly minimal intratumoral hemorrhage (hemosiderin deposition) [19, 20]. On pathology, we found 10 cases of CCDC whereas only one case of CRCC with intratumoral hemorrhage (hemosiderin deposition) (*P* < 0.0001).

In our study, 30 patients with CRCC and in 2 with CCDC the tumors had a clear boundary (*P* < 0.0001), which was best seen in the delayed phase. Infiltrative growth is a much more common pattern with CCDC where a WEs CRCC tumors grow by radial exogenous appearance [21]. Infiltrative lesions may enlarge the kidney but usually maintain the reniform contour. These growth patterns can often be distinguished on cross-sectional imaging through analysis of tumor morphology [22]. As evident in our series, both infiltrative and exogenous patterns may coexist to various degrees.

In 31 patients with CRCC and in 2 with CCDC the tumors had thickened enhancing internal septations [23, 24]. Specific lesions that can look like CRCC or CCDC [25] include some hemorrhagic cysts, atypical cysts, cystic nephroma, and extensively CRCC [26]. Cystic nephroma consists of a circumscribed mass of cysts with intervening fibrous septa, occasionally areas of calcification [27]. Unlike in multilocular CRCC, the cyst lining and septa do not exhibit evidence of clear cell proliferation [28].

In one patient with CRCC and in 10 with CCDC the tumors invaded the renal pelvis or ureter (*P* < 0.0001). In 2 patients with CRCC and 10 patients with CCDC had retroperitoneal lymph node or distant metastasis (*P* < 0.0001). Hence the differential biological behavior of the tumors may also provide useful diagnostic information [29].

In the preoperative radiological work-up, MRI is the best modality for providing important information to diagnose RCC subtypes [30]. In our study, all CRCCs appeared hypointense on T1-weighted images and hyperintense on T2-weighted images, however, all CCDCs appeared hypointense on T1-weighted images and hypointense on T2-weighted images(*P* < 0.0001). In general, CRCC appears slightly hypointense to normal renal parenchyma on T1-weighted images and hyperintense on T2-weighted images. The CCDC was hypointense on T2-weighted images and did not have a hypointense rim. Along with MRI, hypointensity on T2-weighted images appears to favor CCDC, especially with a centrally located tumor.

Renal cell carcinoma, the most common neoplasm of the adult kidney accounts for 2–3 % of all malignant diseases in adults. Although imaging techniques for abdominal screening have led to the increased incidental detection of renal tumor, unfortunately 25–30 % patients still have metastases at presentation. Metastatic renal cell carcinoma is one of the most treatment resistant malignancies and patients have a dismal prognosis with a < 10 % 5-year survival rate. The identification of markers that can predict the potential of metastases will have a great impact in improving the patient’s outcome. The described monoclonal antibody might be used as a research tool to assess bilitranslocase as a marker of transition from normal tissue to its neoplastic transformation in human kidney [31].

Information regarding the clinical behavior of these two tumors is limited due to its rare incidence. Among our 33 patients with CRCCs, they were alive without manifesting disease or any other signs or symptoms from 3 years to10 years follow-up. However, 13 patients with CCDCs, 9 patients expired within 5 years of the initial diagnosis and the others are currently still alive. Although the clinical course of these patients is rather indolent, routine follow-up is still mandatory. The correct distinction of the tumors can lead to better understanding of their clinicopathologic differences, which should aid in developing individualized management plans.

Our study has several limitations. First, our study is limited by the relatively small number of patients with the two rare tumors. Further research is needed to verify our findings in larger patient populations. Second, only13 patients with CRCC and 4 patients with CCDC underwent MR examinations, so, the MR features of these two renal tumors need further investigation. Third, standardized definitions of imaging features of renal masses was not used. Fourth, the retrospective nature and single center of this study might have introduced some form of patient selection bias, therefore, prospective and multi-center studies are recommended.

## Conclusions

In conclusion, distinguishing features of CRCC and CCDC included calcifications, capsule signs, infiltrative appearance, metastasis, internal septations, mural nodules and signal on CT or MR images. These imaging features may help in differentiating the two renal tumor types.

## Data Availability

The image dataset is available at the Medical Imaging, Clinical Medical College, Yangzhou University, Yangzhou, China.

## Abbreviations

CRCC: Cystic renal cell carcinoma
CCDC: cystic collecting duct carcinoma
MRI: Magnetic resonance imaging
ROI: Region of interest
